# Factors affecting decisions of an HMO Drug Exemptions Committee on individual patient requests for coverage of non-formulary drugs

**DOI:** 10.1186/s13584-024-00617-9

**Published:** 2024-07-15

**Authors:** Yael Topol, Lior Weiss, Yossi Lomnicky, Inbal Yifrach-Damari, Noa Markovits, Ronen Loebstein, Itai Gueta

**Affiliations:** 1 Department of Pharmacy and Clinical Pharmacology, Health Division, Maccabi Healthcare Services, Tel Aviv, Israel; 2 Institute of Clinical Pharmacology, Sheba Medical Center, Tel Hashomer & Sackler School of Medicine, Tel Aviv University, Tel Aviv, Israel; 3 Department of Internal Medicine A, Sheba Medical Center, Tel Hashomer, Israel

**Keywords:** Decision making, Drug formulary committee, HMO, Health policy

## Abstract

**Background:**

In Israel, coverage of health needs is delivered by four health maintenance organizations (HMOs), which are budgeted by the government according to the recommendations of the National Drug Formulary (NDF) Committee. For medications not listed in the NDF, individuals may request to cover the costs by the HMO Exemptions Committee (DEC). The objectives of the current study, a first of its kind, are to document the DEC decision process, to identify its components and to determine the decisions’ clinical outcome.

**Methods:**

This retrospective cohort study included all members (≥ age 18) of the Maccabi Healthcare Service (MHS) who submitted a request to the DEC between June 2017 and December 2018. Collected data include patient demographics, clinical information and components of the decision process. Decision success (i.e., clinical outcome correlated with DEC decision) was determined by clinical outcome over at least one-year follow-up.

**Results:**

A total of 335 requests were included. Strong evidence and rare disease were positively associated with approvals, while the availability of alternative treatments and costs were negatively associated. The majority of decisions (75%) met predicted clinical outcomes. Only estimated costs were found to be associated with decision success.

**Conclusions:**

Factors that reduce the potential costs of a requested drug are significantly associated with higher odds for drug approval, but only when the evidence supports potential benefit.

**Supplementary Information:**

The online version contains supplementary material available at 10.1186/s13584-024-00617-9.

## Introduction

In the current climate of worldwide mounting drug costs and insufficient financing, health policy decision makers, at both the governmental level and healthcare delivery organizations, are tasked with defining feasible criteria for equitable prioritization of resource allocation [1, 2]. In Israel, coverage of health needs of all resident Israelis, as defined by the National Health Insurance Law of 1995 (NHIL), is delivered by four Health Maintenance Organizations (HMOs), among which Maccabi Healthcare Services (MHS) is the second largest, covering approximately 2,700,000 people.

Government budgeting for HMO prescription medication costs is updated annually as part of the National Drug Formulary (NDF) by the interdisciplinary NDF Committee (NDFC). Nevertheless, the NDF cannot accommodate all FDA approved medications or those used off-label. Examples include the off-label evidence-based use of eculizumab, a C5a inhibitor, for CHAPLE syndrome; the inclusion of sacubitril-valsartan for heart failure cases with 35% ejection fraction cutoff; or the inclusion of dupilumab indicated for atopic dermatitis only as second line systemic treatment and following treatment with conventional agents (methotrexate, cyclosporine) despite the former being considered first-line systemic treatment, but given the latter lower costs and reasonable efficacy [3, 4].

To overcome these barriers, each HMO may also offer supplementary insurance, covering a variety of healthcare needs, including requests for funding of drugs not included in the NDF [5]. Individual patient requests for an HMO to cover costs of a drug not included in the NDF must be referred to its mandatory HMO Drug Exemptions Committee (DEC), which was established by the HMO, regularized by the Ministry of Health (MOH) in 2010 and further characterized taking into account several case-laws [6–8].

The Maccabi DEC is an interdisciplinary group of 12 members, each with an equal vote: 3 pharmacists, 6 expert physicians, a social worker, a legal advisor and a budget advisor, supported (upon request) by nonmember experts, covering all medical specialties. The decision-making process for approval or denial of a request is based on assessing the components provided by the MOH (e.g., availability of alternative treatment, case uniqueness, etc.) which are detailed elaboration of the components proposed by D. M. Eddy in 1990, restated in 2007 and “formalized” (in the U.S.) more recently, namely, scientific evidence, expected clinical outcomes, and impact on policy decisions – including budgeting, legal ramifications and ethical (personal) value judgments [2, 6, 9–13].

While several previous publications have reported the rate of requests approved by the DECs, none have assessed the relative contribution of each component to the final decision nor the decision’s clinical outcome [6, 14]. The objective of the current study was to document the decisions (approval or denial) of an HMO DEC operating under strict mandatory rules, to identify the components of the decision process affecting approval or denial, to determine the proportion of decisions that correlated with the expected clinical outcome, and to identify predictors affecting those outcomes. We hypothesized that (1) approvals are associated with requests supported by scientific evidence, no available alternative treatment and lower expected costs implications, and that (2) decision’s clinical outcomes are mainly associated with the scientific evidence.

## Methods

### Study design and cohort definition

A retrospective cohort study was conducted using the MHS computerized database, which maintains longitudinal data on all its insureds. In this database, data are automatically collected and include all laboratory results from a single central lab, full pharmacy prescription and purchase data, drugs dispensed and extensive patient-level information, including demographic variables, outpatient clinic physician visits, hospital admissions, selected diagnoses listed in automated generic, algorithm validated disease registries drawing upon ICD-9-CM coding, physician entered diagnoses, hospital discharge codes and billing information from providers. The database is used for pharmaco-epidemiological research [15]. The study protocol was approved by the Maccabi Institutional Review Board (reference number 142-20-MHS), which waived the requirement of informed consent contingent on de-identified data. All methods were performed in accordance with statement for observational the ethical standards as laid down in the Declaration of Helsinki and its later amendments [16].

### Cohort and covariates

The study cohort comprised all individuals ≥ 18 years old, for whom a decision regarding application for a drug not budgeted by the NDF was issued by the DEC between June 1, 2017, and December 31, 2018. All individuals were followed for at least one year after the decision date. Medication requests for hemato/oncological indications were excluded (60% of total requests), as serial published government yearly reports have shown that over 50% of annual supplements to the NDF budget are regularly preallocated for hemato/oncology drugs. This is probably due to strong public advocacy demands, likely driven by pharmaceutical industry advertising and possible influence on expert opinion leaders, who participate ex-officio in national policy making, as shown in other countries [17–20].

For each submitted request, data were collected on patient demographics (gender, age), submitting physician (GP vs. specialist), and components of the decision process itself, recorded and dichotomously categorized as follows:

1. Strength of published evidence: weak vs. moderate or strong (case reports or series vs. RCTs, meta-analyses).
2. Rare disease: not rare vs. rare (documented incidence < 1:100,000/patient).
3. Unique case: not unique vs. unique (characterized by additional comorbidities with possible benefit for the requested medication or prohibiting use of any reasonable alternatives).
4. Alternative treatment availability: no vs. yes (whether alternative evidence-based treatment of comparable effectiveness and tolerability is available).
5. Urgency: no vs. yes (need for immediate treatment, e.g., life-threatening conditions, vital organs at risk)
6. Estimated budget impact: above vs. below the management policy limit (calculated for one year of treating all patients with the same medical profile).
7. Organization policy: pertinent vs. not pertinent (legal precedents, optional supplemental MHS HMO insurance covering request).
8. Ethical preferences: Decision unanimous vs. majority decision with documentation of individual reasons for abstention or objection, recorded as minority opinions.

Decision clinical success was defined according to its clinical outcome, as judged by two independent reviewers (YT and LW) following medical record review. Disagreements were solved by consensus:

**A match: DEC decision met predicted clinical outcome**:

1. Requested drug was approved, followed by significant clinical benefit (i.e., survival, reduced health care utilities and improved disease markers).
2. Requested drug was denied, followed by significant clinical benefit without the requested treatment or drug obtained with private insurance, followed by no clinical benefit.

**A mismatch: DEC decision did not meet predicted clinical outcome**:

1. Requested drug was approved, followed by no clinical benefit, or was associated with significant predictable adverse events leading to drug discontinuation or in cases of no adherence.
2. Requested drug was denied, followed by clinical deterioration or drug obtained with private insurance followed by significant clinical benefit.

## Data analysis

Comparisons between groups were conducted using the Mann‒Whitney U test for continuous variables and the chi-square test or Fisher’s exact test for categorical variables. Data are presented as the mean ± standard deviation (SD) or median with interquartile range (IQR) as appropriate for continuous variables and proportions for categorical variables, respectively. Univariate analysis was employed to evaluate the association between the various variables and each outcome (approval vs. rejection and match vs. mismatch). Variables found to be significantly associated with the outcome were further analyzed by adjusted logistic regression. To isolate the effects of costs on decision appropriateness, we conducted a sensitivity analysis including only those cases entailing estimated costs lower than the limit dictated by management. All analyses were two-tailed, and a *p* value ≤ 0.05 was considered significant. Statistical analyses were performed using SPSS software (version 25).

## Results

Overall, 369 relevant requests were submitted for discussion between June 1, 2017, and December 31, 2018. Of these, 24 did not meet submission requirements, and 10 were excluded due to incomplete follow-up information. Of the included 335 requests, 282 (84.2%) were approved, and 53 (15.8%) were denied (drug names and indications can be found in Table S1 in supporting information). Patients were followed for a median of 33 months (range: 20–40). On follow-up, clinical outcomes among 215/282 (76.2%) approvals and 25/53 (47.2%) denials indicated a match with the DEC decision (*p* < 0.001).

Univariate analysis showed that strength of evidence, rare disease, unique case, lack of alternative treatments, specialist recommendation and annual costs below management limit were significantly associated with approvals (Table 1). Most decisions were unanimous (rates 98% vs. 96% for approvals and denials, respectively). Multivariate logistic regression, after ascertaining the absence of collinearity, demonstrated that strength of evidence (OR 40.7, 95% CI 7-237) and rare disease (OR 7.2, 95% CI 1.1–46.7) were positively associated with approvals. The availability of alternative treatments (OR 0.2, 95% CI 0.1–0.5, *p* < 0.001) and costs above the management limit (OR 0.01, 95% CI 0.00-0.04) were negatively associated with approvals (Table 2).

**Table 1:** Case characteristics by approvals and denials (*n* = 335)

	Approvals	Denials	*P* value
N	282 (84.2%)	53 (15.8%)	
Age, years (SD)	46 (± 23)	48 (± 22)	0.586
Gender, F	148 (52.5%)	25 (47.2%)	0.478
Urgency	28(9.9%)	3(5.7%)	0.442
Strength of evidence: Moderate or strong	269 (95.4%)	46 (86.8%)	0.025
Rare disease	46 (16.3%)	2 (3.8%)	0.017
Unique case	116 (41.1%)	9 (17%)	0.001
No alternative treatments	191 (67.7%)	12 (22.6%)	< 0.001
Excessive estimated costs	31 (11%)	45 (84.9%)	< 0.001
Supplemental HMO insurance	16 (76.2%)	5 (23.8%)	0.349
Specialist referral	255 (90.4%)	38 (71.7%)	< 0.001
Clinical outcome indicates a match with DEC decision	215 (76.2%)	25 (47.2%)	< 0.001

**Table 2:** Adjusted logistic regression for approvals

Variable	OR	95% CI	*P* value
Age	1.02	0.99–1.04	0.13
Strength of evidence: Moderate or strong	40.69	6.99-236.95	< 0.001
Rare disease	7.19	1.11–46.64	0.04
Unique case	1.21	0.35–4.20	0.76
Alternative therapies	0.20	0.08–0.48	< 0.001
Excessive estimated costs	0.01	0.00-0.04	< 0.001
Submitting physician: specialist	1.05	0.36–3.04	0.93

Of the denials with moderate-to-high level of evidence, 37/46 (80.4%) requests had an alternative treatment available in the NDF, 41/46 (89.1%) and 45/56 (97.8%) cases were defined as not unique or rare, respectively, and 43/46 (93.5%) had an estimated costs above the management policy limit.

With respect to clinical outcomes, only estimated costs were found to be associated with decision success: Decisions defined as mismatch showed significantly higher rates of requests for which costs were higher than the limit dictated by management (Table 3). Sub analysis in which only cases with lower than limit potential costs were included (*n* = 259) did not change the results: none of the variables was associated with decision clinical outcome, the majority of decisions (75% in either approved or declined) matched the clinical outcome during ≥ 1 year follow-up, and 25% of declines did not. Conversely, among those cases with higher costs, only 47% of those who were declined were found to match the expected clinical course (*p* < 0.001).

**Table 3:** Univariate analysis by decision clinical outcome

	Clinical outcome indicates a:		
	Match	Mismatch	*P* value
N	240	95	
Age	46 (± 23)	48 (± 22)	0.631
Gender, F	132 (55%)	41 (43.2%)	0.051
Urgency	24 (10%)	7 (7.4%)	0.454
Strength of evidence: Moderate or strong	225 (93.8%)	90 (94.7%)	0.731
Rare disease	36 (15%)	12 (12.6%)	0.577
Unique case	92 (38.3%)	33 (34.7%)	0.540
No alternative treatments	91 (37.9%)	41 (43.2%)	0.376
Excessive estimated costs	45 (18.8%)	31 (32.6%)	0.006
Supplemental HMO insurance	15 (6.3%)	6 (6.3%)	0.982
Specialist referral	211 (87.9%)	82 (86.3%)	0.690

## Discussion

Previous publications have outlined how HMO DECs should function and several have also addressed the rate of approval or denial by such committees [6, 14, 21, 22]. However, no study has examined the relative contribution of the various components in the decision-making process of these committees, nor the impact of the decisions on the subsequent clinical course during follow-up.

The process of decision-making comprises two main steps, aiming to deliver the best clinical outcome in resource-limited health-care systems: analyzing and estimating outcomes (clinical and financial) of all available practices, followed by comparing all possible options that eventually lead to a decision. While the former step – as described by Eddy – is a question of facts, the latter is a question of personal values and preferences. Our findings, in which more requests were approved and the majority met the predicted clinical outcome (i.e., a match), are in line with Eddy’s model (proposed in the U.S. but yet untested there or elsewhere), further reinforces MOH guidelines and our DEC current decision-making process.

This study shows that factors that enable one request to be distinguished from a large group of similar potential requests (e.g., case uniqueness, rare disease, no alternative therapy) and those lowering estimated annual costs were independently associated with approvals. Importantly, published evidence supporting potential effectiveness remained significant in both uni- and multivariate analyses, reflecting the DEC core principle of practicing evidence-based medicine. However, these findings, as suggested in Eddy’s second step, did not assure the clinical success of the decisions. Accordingly, we sought to identify factors associated with decision clinical outcomes.

Univariate analysis did not identify quantitative factors associated with decision outcomes other than excessive estimated costs. However, following median of 2.5 years follow-up, more than 70% of decisions were found to have the anticipated clinical outcome. Overall, we believe that these findings reflect Eddy’s second step in the process of decision-making: a step characterized by personal values and organizational preferences. Unfortunately, the latter was dictated mainly by excessive estimated costs, as shown by the higher rate of decisions that were defined as mismatches.

Personal values are difficult to quantify, particularly given the heterogeneity of patient characteristics and drugs requested, and yet nearly all decisions were unanimous. Behavioral economists studying decision-making have identified cognitive biases, which are psychological tendencies to process information in predictable patterns. These cognitive biases can lead to errors or irrational decisions, which in our case might be affected by the main disciplines of the committee members. Studies suggest that errors such as framing effect and delay discounting may be common among patients and providers when making health care decisions [23]. Other factors that may affect decision-making include professional background and experience, activity sector and gender [24, 25]. In fact, the majority of decisions correlated with the anticipated clinical outcome. We believe that the multidisciplinary professionals comprising the DEC, their varying expertise and clinical experiences, their wide age range (37–83), position outside of the DEC (senior physicians, head of hospital ward, managers in healthcare providers, health policy makers, etc.), along with each member’s equal vote, may by themselves be important determining factors for decision success. Hence, such a bias in the current DEC format is probably of little significance.

Several limitations should be noted. First, the retrospective nature of the study prevented the assessment of patients lost to follow-up or of the evaluation of reasons for noncompliance. We also excluded all requests for drugs indicated for hemato-onco- and oncology conditions; hence, our findings cannot be generalized for this group of patients. Additionally, some of the variables were dichotomously categorized (e.g., strength of evidence); Given that the DEC deals with cases that can often be considered medical outliers, evidence regarding treatment efficacy or toxicity is often scarce. Hence, the thumb rule is that in such cases, even weak evidence can be acceptable. This approach might also be relevant to other decision process components (e.g., case uniqueness, availability of alternative treatments) as well as to the many aspects related to value judgment. This latter component comprises many faces and layers, both organizational and personal, that could not be comprehensively covered. Future studies should address the different aspects and impact of personal values in the process of decision-making in such committees. Given the lack of a comparator group (such as a smaller DEC), we could not evaluate the contribution of committee size and composition (i.e., number of members and their disciplines).

## Conclusions

Our findings suggest that factors enabling the reduction of estimated costs based on either patient or both drug characteristics are associated with higher odds for request approvals but not at the expense of adherence to evidence-based rules. Future studies should evaluate the contribution of the committee’s heterogenicity itself to the decision’s clinical outcome.

### Electronic supplementary material

Below is the link to the electronic supplementary material.

Supplementary Material 1

## Data Availability

The datasets and analyzed during the current study available from the corresponding author on reasonable request.

## Abbreviations

DEC: Drug Exemptions Committee
HMO: Health maintenance organization
MHS: Maccabo Healthcare Services
MOH: Ministry of Health
NDF: National Drug formulary
NDFC: National Drug Formulary Committee
