# Supplementary material for: Factors affecting decisions of an HMO Drug Exemptions Committee on individual patient requests for coverage of non-formulary drugs
Source: Isr J Health Policy Res. 2024 Jul 15;13:30. doi: 10.1186/s13584-024-00617-9 (PMC11247773; doi:10.1186/s13584-024-00617-9)
Supplement: Supplementary file 1 — Supplementary Material 1 [file 13584_2024_617_MOESM1_ESM.pdf]

Tasble S1. List of requested drugs and indications during study period

Drug (brand name)	Generic name	Indication	% approved per active ingredient
ABBOSYNAGIS	RSV vaccine	Pyriform Stenosis	100%
ACLASTA	zoledronic acid	Osteopenia	100%
ACTEMRA	tocilizumab	Systemic sclerosis	100%
ACTEMRA	tocilizumab	Takayasu disease	
AMBISOME	amphotericine B	Allergic bronchopulmonary aspergillosis (cf)	100%
AREDIA	pamidronate	Erdheim Chester Disease	100%
ARIXTRA	fondparinux	Deep Veins Thromboembolism	100%
ARIXTRA	fondparinux	Cerebral Artery Thrombosis	
ATTENT	mixed amphetamine	ADHD	100%
AVASTIN	bevacizumab	Recurrent respiratory papilomatosis	100%
BELVIQ	lorcaserin	Dravet Syndrome	100%
BERINERT	C1 esterase inhibitor	Hereditary Angioedema	100%
BOTOX	onabotulinumtoxinA	Hyperactive Bladder	94%
BOTOX	onabotulinumtoxinA	Bell's Palsy	
BOTOX	onabotulinumtoxinA	Frey's Syndrome	
BOTOX	onabotulinumtoxinA	Multiple Sclerosis	
BOTOX	onabotulinumtoxinA	Hypophonia Post Laryngectomy	
BOTOX	onabotulinumtoxinA	Anal Fissure	
BOTOX	onabotulinumtoxinA	Drooling	
BOTOX	onabotulinumtoxinA	Hypertonia	
BOTOX	onabotulinumtoxinA	Spasm	
BOTOX	onabotulinumtoxinA	Pylorospasm	
BOTOX	onabotulinumtoxinA	Pharingeal Fibromatosis	
BOTOX	onabotulinumtoxinA	Spastic Hemiparesis	
BOTOX	onabotulinumtoxinA	Cerebral Palsy	
BOTOX	onabotulinumtoxinA	Pachyonychia Congenita	
BOTOX	onabotulinumtoxinA	Interstitial Cystitis	
BOTOX	onabotulinumtoxinA	Parkinson Disease	
BRILINTA	ticagrelor	Post PCI	100%
BRILINTA	ticagrelor	TIA	
BRILINTA	ticagrelor	Peripheral Vascular Disease	
BRILINTA	ticagrelor	Unstable Angina Pectoris	
BRIVIACT	brivaracetam	Epilepsy	100%
CAYSTON	aztreonam	CF - continuous tretment	100%
CINACALCET-TEVA	cinacalcet	Primary Hyperparathyroidism	100%
CINQAIR	reslizumab	Churg-Strauss Syndrome	100%
COPAXONE	glatiramer	Multiple sclrosis remitting relapsing	50%
COPAXONE	glatiramer	Multiple sclerosis primary progressive	
COSENTYX	secukinumab	Ankylosing spondylitis	50%
COSENTYX	secukinumab	Psoriatic arthritis	
DAKLINZA	daclatasvir	HCV	100%
DEFITELIO	defibrotide	Prophylaxis of hepatic veno-occlusive disease	100%
DYSPORT	abobotulinumtoxinA	Hydronephrosis	100%
EFFIENT	prasugrel	CVA	100%
ELIQUIS	apixaban	CVA	93%
ELIQUIS	apixaban	Thrombophilia Protein S Deficiency	
ELIQUIS	apixaban	Peripheral Vascular Disease	
ELIQUIS	apixaban	Patent Foramen Ovale	
ELIQUIS	apixaban	Pulmonary Embolism	
ENBREL	etanercept	Chronic Osteomyelitis	100%
ENBREL	etanercept	Behcet's Syndrome	
ENBREL	etanercept	GI Acute GVHD	
ENTRESTO	sacubitril/valsartan	Congestive Heart Failure	100%
ENTYVIO	vedolizumab	Acute GVHD	100%
EPCLUSA	sofosbuvir/velpatasvir	HCV + cryoglobulinemia	100%
EXONDYS 51	eteplirsen	Duchenne mascular dystrophy	0%
EXVIERA	dasabuvir	HCV	0%
EYLEA	aflibercept	Central Serous Retinopathy	
EYLEA	aflibercept	Cystoid Macular Edema	

EYLEA	aflibercept	Choroidal Ocular Hemangioma	67%
EYLEA	aflibercept	Radiation Retinopathy	
EYLEA	aflibercept	Age related Macular Degeneration	
EYLEA	aflibercept	BEST disease	
EYLEA	aflibercept	Coat's Disease	
EYLEA	aflibercept	Retinal Vein Occlusion	
EYLEA	aflibercept	Diabetic macular edema	
FEBURIC	febuxostat	Gout	100%
FERINJECT	ferric carboxymaltose	Budd Chiari Syndrome	100%
FERINJECT	ferric carboxymaltose	Iron Deficiency	
FORTEO	teriparatide	Osteoporosis	95%
FORTEO	teriparatide	Nonunion Fracture	
FORTEO	teriparatide	Hypoparathyroidism	
GENOTROPIN	somatropin	Charge Syndrome	67%
GENOTROPIN	somatropin	Neurofibromatosis type 1	
HEMLIBRA	emicizumab	Factor 8 disorder	100%
HUMIRA	adalimumab	Folliculitis decalvans	100%
HUMIRA	adalimumab	Vasculitis (familial Georgian, deficiency of ADA2)	
HUMIRA	adalimumab	Pouchitis	
HUMIRA	adalimumab	Sarcoidosis	
ILARIS	canakinumab	Familial Mediterranean fever	100%
IVIg	IVIg	Motor neuropathy	76%
IVIg	IVIg	Sjogren's syndrome related neuropathy	
IVIg	IVIg	Autoimmune small fiber neuropathy	
IVIg	IVIg	Autoimmune encephalitis	
IVIg	IVIg	Congenital neutopenia	
IVIg	IVIg	Scleroderma	
IVIg	IVIg	Seizures	
IVIg	IVIg	Neuromyelitis optica spectrum disorder	
IVIg	IVIg	Autoimmune hepatitis	
IVIg	IVIg	Systemic lupus erythematosus	
IVIg	IVIg	Pompe's Disease	
IVIg	IVIg	Recurrent abortions	
IVIg	IVIg	Severe neutropenia	
IVIg	IVIg	Small fiber neuropathy	
IVIg	IVIg	Moya Moya Disease	
JAKAVI	ruxolitinib	GVHD	92%
JAKAVI	ruxolitinib	Polycytemia vera	
KALYDECO	ivacaftor	CF, A455E mutation	100%
KALYDECO	ivacaftor	CF, w1282x/D1152H mutations	
KINERET	anakinra	Pericarditis (recurrent)	100%
KINERET	anakinra	Hidradenitis suppurativa	
KINERET	anakinra	FIRES (febrile infection-related epilepsy syndrome)	
LORMYX	rifaximin	Colon Diverticulosis	100%
LORMYX	rifaximin	Irritable Bowel Syndrome	
LUCENTIS	ranibizumab	Central Serous Retinopathy	83%
LUCENTIS	ranibizumab	Choroidal Neovascularization	
LUCENTIS	ranibizumab	Cystoid Macular Edema	
LUCENTIS	ranibizumab	Central Retinal Vein Occlusion	
LUCENTIS	ranibizumab	Age related Macular Degeneration	
LYXUMIA	lixisenatide	Reactive Hypoglycemia	100%
MABTHERA	rituximab	Systemic sclerosis	88%
MABTHERA	rituximab	Cryoglobulinemia - maintenance	
MABTHERA	rituximab	Polyarteritis nodosa	
MABTHERA	rituximab	Churg strauss vasculitis	
MABTHERA	rituximab	Interstitial lung disease (NSIP)	
MABTHERA	rituximab	Multiple sclerosis primary progressive	
MABTHERA	rituximab	Autoimmune encephalitis	
MABTHERA	rituximab	IGG4-related disease	
MABTHERA	rituximab	Susac syndrome	
MABTHERA	rituximab	Pemphigus vulgaris	
MABTHERA	rituximab	Anti phospholipid syndrome	
MABTHERA	rituximab	Systemic lupus erythematosus	

MABTHERA	rituximab	Opsoclonus myoclonus ataxia	
MABTHERA	rituximab	Fibrillary glomerulopathy	
MABTHERA	rituximab	Polymyositis	
MABTHERA	rituximab	Hemophilia (acquired)	
MABTHERA	rituximab	Immune-mediated motor neuropathy	
MABTHERA	rituximab	Glycogen storage disease II (Pompe)	
MABTHERA	rituximab	Multiple sclerosis relapsing remitting	
MABTHERA	rituximab	Nephrotic syndrome (minimal change disease)	
MULTAQ	dronaderone	Ectopic Atrial Tachycardia	0%
NATPARA	parathyroid hormone	Primary hypoparathyroidism	0%
NORDITROPIN	somatropin	Morquio Syndrome	0%
NUCALA	mepolizumab	Hypereosinophilic syndrome	100%
NUCALA	mepolizumab	Asthma	
OCREVUS	ocrelizumab	Multiple sclerosis primary progressive	67%
OCREVUS	ocrelizumab	Multiple sclerosis secondary progressive	
OFEV	nintedanib	Idiopathic pulmonary fibrosis	100%
ORENCIA	abatacept	Scleroderma	100%
ORFADIN	nitisinone	Type 1 albinism oculocutaneous (OCA)	0%
OTEZLA	apremilast	Vitiligo	0%
OZURDEX	dexamethasone	Cystoid Macular Edema	75%
OZURDEX	dexamethasone	Pseudophakic Cystoid Macular Edema	
OZURDEX	dexamethasone	Macular Edema	
PALEXIA	tapentadol	Vulvodynia	100%
PERIACTIN	cyproheptadine	Hemicrania Continua	100%
PRADAXA	dabigatran	Pulmonary Thromboembolism	67%
PRADAXA	dabigatran	Thromboembolism	
PRALUENT	alirocumab	Dislipidemia	50%
PROLIA	denosumab	Osteoporosis	100%
PROLIA	denosumab	Fibrous Dysplasia	
PROLIA	denosumab	Osteopenia	
PROLIA	denosumab	Paget's disease of the bone	
REMICADE	infliximab	Colitis (immunotherapy-induced)	100%
REMICADE	infliximab	Sarcoidosis	
REMICADE	infliximab	Takayasu disease	
REMICADE + ENTYVIO	infliximab + vedolizumab	RA + Crohn's	
REPATHA	evolocumab	Hypercholesterolemia	50%
REVOLADE	eltrombopag	Immune throbocytopenia	100%
REVOLADE	eltrombopag	Aplastic anemia	
SANDOSTATIN LAR	octreotide	Chylous ascites	100%
SANDOSTATIN LAR	octreotide	Dumping syndrome postgastrectomy	
SATIVEX	nabiximols	Tourettes Disorder	50%
SATIVEX	nabiximols	Spasticity	
SAXENDA	liraglutide	Obesity	80%
SIMDAX	levosimendan	Congenital Chronic Heart Failure	100%
SIMPONI	golilumab	Crohn's Disease	50%
SIMPONI	golilumab	Ulcerative colitis + polyarthralgia	
SOLIRIS	eculizumb	HUS-like	100%
SOLIRIS	eculizumb	atypical HUS-like	
SOLIRIS	eculizumb	Chaple syndrome	
SOLIRIS	eculizumb	Degos disease	
SOVALDI	sofosbuvir	HCV	100%
SPINRAZA	ataluren	Spinal muscular atropthy	0%
STELARA	ustekinumab	Crohn's disease	75%
STELARA	ustekinumab	Ulcerative colitis + psoriasis	
STRATTERA	atomoxetine	Attention deficit disorder, Tics	0%
TENOFOVIR	tenofovir	Hepatitis B	100%
THALIDOMIDE	thalidomide	Behcet's Syndrome	100%
TRULICITY	dulaglutide	Diabetes type 2	67%
TRULICITY	dulaglutide	Hypothalamic Obesity	
VICTOZA	liraglutide	Diabetes type 1	67%
VIEKIRAX	Ombitasvir/paritaprevir/ritonavir	HCV	0%
VYVANSE	lisdexamphetamine	Attention deficit hyperactivity disorder	75%
VYVANSE	lisdexamphetamine	Narcoplesy with cataplesy	
XARELTO	rivaroxaban	Anti phospholipid syndrome	

XARELTO	rivaroxaban	Peripheral vascular disease	90%
XARELTO	rivaroxaban	Mural thrombus of the heart	
XARELTO	rivaroxaban	Deep veins thrombosis	
XARELTO	rivaroxaban	Superficial vein thrombosis	
XELJANZ	tofacitinib	Ankylosing Spondylitis	63%
XELJANZ	tofacitinib	Ulcerative Colitis	
XELJANZ	tofacitinib	Vitiligo	
XELJANZ	tofacitinib	Alopecia areata	
XGEVA	denosumab	Hypercalcemia	80%
XGEVA	denosumab	Bone metastases	
XOLAIR	omalizumab	Bollous pemphigoid	72%
XOLAIR	omalizumab	Chronic urticaria	
XOLAIR	omalizumab	Asthma	
XOLAIR	omalizumab	Chronic idiopathic urticaria	
XULTOPHY	degludec/liraglutide	Diabetes type 2	100%
ZEBINIX	eslicarbazepine	Epilepsy	100%
ZEPATIER	elbasvir/grazoprevir	HCV	0%
ZONISAMIDE	zonisamide	Lewy body disease	0%
